# Association between air quality satisfaction, family relationships, and depression symptoms among middle-aged and elderly chinese people: the mediation role of perceived health status

**DOI:** 10.1186/s12889-022-14711-7

**Published:** 2022-12-27

**Authors:** Zhiping Niu, Mengxi Zhai, Yu Dong, Weihong Wen, Lina Xue, Maieryemuguli Aosiman, Weijun Qin, Zhizhou Duan

**Affiliations:** 1 Department of Urology, Xijing Hospital, The Fourth Military Medical University, 127 West Changle Road, Xi’an, China; 2 School of Public Health, Wuhan University, Wuhan city, China; 3 Department of Epidemiology and Health Statistics, School of Public Health, Southeast University, Nanjing, China; 4 Institute of Medical Research, Northwestern Polytechnical University, 127 Youyi Road, Xi’an, China; 5 Department of Medical Affairs, Tangdu Hospital, Fourth Military Medical University, 1 Xinsi Road Baqiao District, Xi’an, China; 6 Preventive Health Service, Jiangxi Provincial People’s Hospital, The First Affiliated Hospital of Nanchang Medical College, Nanchang, Jiangxi China

**Keywords:** Depression symptom, Air quality satisfaction, Family relationship, Perceived health status, Mediation analysis

## Abstract

**Background:**

Population aging has led to depression becoming a serious public health problem both in China and worldwide. Marital relationships, relationships with their children, and air pollution might play an important role in the process of depressive disorders. In this study, we aimed to reveal the mechanism of the effects of these factors on depression.

**Methods:**

Participants were recruited from The China Health and Retirement Longitudinal Study (CHARLS) (wave 4) from July 2018 to March 2019. Depression symptoms were evaluated using the 10-item Center for Epidemiologic Studies depression scale (CESD-10). Marital relationships, relationships with their children, air quality satisfaction, and perceived health status were analyzed using Likert 5-point evaluation methods. Structural equation modeling-path (SEM) models were used to explore these variables’ mediation effects on depression symptoms.

**Results:**

Marital relationships, relationships with their children, air quality satisfaction, perceived health status, and depression symptoms were significantly associated with each other (*P* < 0.001). Mediation analysis showed that family relationships (standardized beta = −0.28 [−0.31, −0.26]) and quality satisfaction (standardized beta = −0.03 [−0.05, −0.01]) had negative effects on depression symptoms. The total indirect effects of family relationships and air quality satisfaction on depression symptoms were −0.06 (95% confidence interval (CI) = [−0.07, −0.05]) and −0.016 (95% CI = [−0.02, −0.01]), respectively.

**Conclusion:**

Family relationships, air quality satisfaction, and perceived health status influenced depression symptoms. The effects of family relationships and air quality satisfaction on depression symptoms were significantly mediated by perceived health status. Therefore, perceived health status aspects should be considered when conducting targeted intervention toward depression symptoms among middle-aged and elderly adults.

**Supplementary Information:**

The online version contains supplementary material available at 10.1186/s12889-022-14711-7.

## Introduction

Population aging is occurring worldwide and has become a focus of public life during the 21st century, particularly in high-income countries such as Japan [1]. The number and proportion of middle-aged and elderly people has been increasing worldwide [2]. As a middle-income country, China is also experiencing population aging. According to China’s National Bureau of Statistics, the number of people aged 60 years old or older reached 253.88 million, with a proportion of 18.1% among all Chinese people [3, 4]. It is estimated that the number of people aged 60 year old or older will be over 400 million by 2050 [5], which will lead to increased social pressure and public health problems.

Depression is an serious public health concern in aging populations [6]. Individuals tends experience increased depression symptoms from middle to older age, which affects their physical health [7, 8]. The World Health Organization stated that over 10% of elderly people had depressive symptoms, which was a prominent cause of physical disease, leading to an increased global health burden [9]. Previous studies showed that the proportion of elderly people with depression symptoms has reached 23.6% in China, which is higher than in other age groups [10]. Depression has become the fourth highest factor causing disabilities, which has increased China’s disease burden [11]. Therefore, depression symptoms should receive more in depth attention among the middle-aged and elderly populations .

Depression symptoms are signs of emotional disorder, and are affected by family and environmental factors [12–16]. For example, Qianrong et al. investigated 139 older couples and found that marital satisfaction of married men could decrease the depression score, suggesting that marital satisfaction was a considerable factor affecting depression in the older population [17]. In addition, the mental health of the elderly is influenced by their relationship with their children [18–20]. For example, Morgan surveyed 19 depressed mothers and found that a poor mother-child relationship was associated with the frequency and severity depression symptoms [20]. Moreover, people’s mental health might be affected by air quality conditions. Environmental studies have shown that people living in areas with high levels of air pollution were more likely to suffer depression [21, 22]. For example, Yao investigated 15,105 middleaged and elderly people and found that exposure to higher PM_2.5_ (particulate matter < 2.5 μm in diameter) increased the risk of developing depression symptoms [22]. Perceived air quality is also an important predictive factor of depressive symptoms that is often ignored [23]. Several studies have indicated that self-reported health status (mainly disease severity and depression) is associated with perceived air quality rather than objective air pollution [23–26]. Therefore, we hypothesized that marital satisfaction, satisfaction with their relationships with their children, and air quality satisfaction would affect the risk of depression.

In addition, perceived health status represents a comprehensive evaluation of people’s own health status, which is influenced by family and environmental factors, playing an important role in the development of depression. Family relationships consist of interparental relationships and family harmony [27–29]. Influenced by traditional culture, martial relationship are an integral part of family harmony in China [30]. Thus, marital relationships and relationships with their children are important dimensions of family relationships and have been recognized as key protective factors of perceived health status among elderly adults [12, 31]. It has been confirmed that negative family relationships can increase the risk of poor health [32].

Moreover, several studies have shown that air quality satisfaction is related to perceived health status [33, 34]. In addition, perceived health status was reported as the most powerful predictor of depressive symptoms [35, 36]. For example, Bae’s research demonstrated that perceived health status and family relationships were the most powerful and key predictors of depressive symptoms among elderly adults [35]. Perceived health status has also been regarded as a mediator of environmental pollution and health [37–39]. For example, Fan’s study suggested that self-rated health played an important mediation role in the association between environmental pollution and subjective well-being [33]. Based on the above evidence, we hypothesized that perceived health status has important mediation effects on the relationships between family relationship satisfaction and air quality satisfaction and the risk of depression.

**Fig. 1:**
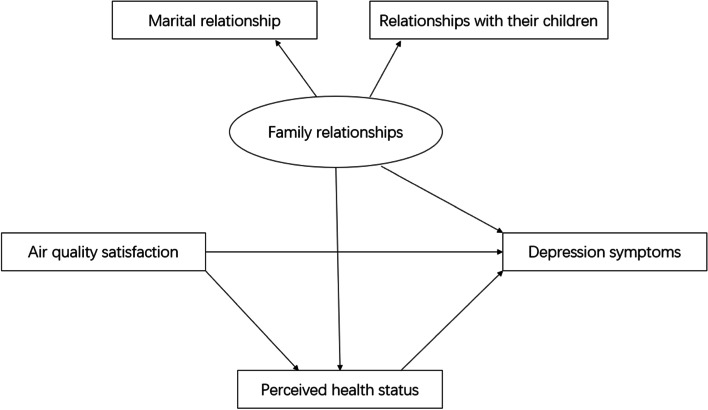
The conceptual model for whole sample, based on a previous study

Depression symptoms are complex and are affected by multiple factors. The mechanism of the effects between family relationships, air population satisfaction, and depression are unclear, representing a barrier to effective prevention. Thus, in the present study, we aimed to explore the mechanism of the effects of these relationships using structural equation models. Our study focuses on the associations of marital relationships, relationships with their children, and air population satisfaction with depression and the mediation effects of perceived health status on these relationships. The conceptual model is shown in Fig. 1.

## Methods

### Samples

All participants were selected from The China Health and Retirement Longitudinal Study (CHARLS), which aims to determine the basic social, economic, and health status characteristics of Chinese people aged 45 years or older. We used the fourth national survey data from December 2020, which was collected between July 2018 and March 2019. For more details please see other research related to CHARLS [33, 40]. Detailed information has been collected at the website (http://charls.pku.edu.cn/). Informed consent was obtained from all participants and the use of CHARLS was approved by the Institutional Review Board of Peking University (approval number: IRB00001052-11015).

In this study, the inclusion criteria were: (1) Complete basic socio-demographic characteristics were available, particularly for age and sex; and (2) full data were available for marital relationships, relationships with their children, perceived health status, air quality satisfaction, and the 10-item Center for Epidemiological Studies Depression Scale (CESD-10). Based on these criteria, a total of 14,148 individuals were enrolled in this study from among 17,708 samples in the database.

### Key variable measures

Marital relationship was measured using the item “How satisfied are you with your marriage (relationship with spouse)”; relationship with their children was assessed using the question “How satisfied are you with your relationship with your children”. Marital status and relationships with their children are representative of family relationships in China [27–30], and we considered family relationships as our unobservable variable when performing statistical analysis in this study. Air quality satisfaction was measured using “How satisfied are you with the air quality this year”, the answers to which ranged from not at all satisfied (scored 1) to completely satisfied (scored 5). Perceived health status was assessed using the item “Would you say your health is very good, good, fair, poor, or very poor”, and a response of “very good” scored 5, whereas “very poor” scored 1.

Depression symptoms were measured using the 10-item 4-Likert CESD-10 scale, which included 8 items related to depression symptoms [41] (e.g., My sleep was restless), and the respondents could choose from “Rarely or none of the time” (scored 0) to “Most or all of the time” (scored 3); the other two items were “I was happy” and “I felt hopeful about my future”, allowing their answers to be calculated numerically. The sum scores were calculated, ranging from 0 to 30, with a higher sum score indicating a higher level of depression symptoms. The cut-off scores were set to 10 and 20 (less than 10, no depression, 10–20, mild depression, > 20, serious depression) respectively [42]. This scale has been confirmed to have good validity and reliability in Chinese elderly populations [43]. In this study, the Cronbach alpha score for internal consistency was 0.80.

### Statistical analysis

The basic socio-demographic variables were descried by the mean ± standard deviation (SD) (for continuous variables) and N/% (for categorical variables). Spearman correlation analysis was used to examine the associations between marital relationships, relationships with their children, air quality satisfaction, perceived health status, and depression symptoms for non-normally distributed data.

In a secondary analysis, mediation analysis was tested using structural equation modeling-path (SEM) analysis (adopting asymptotically distribution-free methods). This analysis determined whether perceived health status was a mediator of the relationship among family relationships, air quality satisfaction, and depression symptoms. In addition, we controlled for age and sex in this mediation analysis, based on previous studies [16, 44, 45]. The model fit index of the mediation analysis indicated a goodness-of-fit of the model, as follows: relative Chi-squared (χ^2^/df) < 5; Comparative fit index (CFI) > 0.90; Tucker-Lewis fit index (TLI) > 0.90; Normed fit index (NFI) > 0.9; Relative non-centrality index (RFI) > 0.9; and the root mean-square error of approximation (RMSEA) < 0.08. Bootstrap methods were used to test the total effect and indirect effect size and their 95% confidence interval (CI). We added other basic socio-demographic characteristics as our control variables for sensitivity testing (age, sex, education level, ethnicity, and residence characteristics) to examine whether perceived health status acted a potential mediator. The statistical analysis was performed using SPSS version 19 (IBM Corp., Armonk, NY, USA) and AMOS 20.0. A *P*-value of less than 0.05 (two tailed) indicated statistical significance.

## Results

A total of 14,148 middle aged and older adults were enrolled in this study. As shown in Table 1, the average age of the participants was 60.23 years (SD = 8.97), and most individuals (72.7%) were 50–69 years old. Among the participants, 92.7% (*n* = 13,112) were of Han ethnicity and 50.2% (*n* = 7100) were male. In addition,72.4% (*n* = 10,239) lived in rural areas. Primary school education level was the highest 43.9% (*n* = 6206) and only 2.3% (*n* = 325) of individuals achieved college education or above. In addition, 64.5% of the participants had no depression symptoms, and 35.5% (*n* = 5024) had depression symptoms. The mean scores of key variables (marital relationships, relationships with their children, air quality satisfaction, perceived health status, and depression symptoms) were 3.43 ± 0.82, 3.64 ± 0.72, 3.16 ± 0.82, 3.11 ± 1.02, 8.21 ± 6.38, respectively.

**Table 1:** The socio-demographic characteristics and key variables in this study

Variables	Total (*N* = 14,148)	Non-depression symptoms (*N* = 9124)	Depression symptoms (*N* = 5024)
Age (Mean ± SD)	60.23 ± 8.97	60.03 ± 9.04	60.60 ± 8.83
45~	1604 (11.3)	1129 (12.4)	475 (9.5)
50~	5402 (38.2)	3466 (38.0)	1936 (38.5)
60~	4880 (34.5)	3106 (34.0)	1774 (35.3)
70~	1924 (13.6)	1198 (13.1)	726 (14.5)
80–95	338 (2.4)	225 (2.5)	113 (2.2)
Ethnic			
Han group	13,112 (92.7)	8471 (92.8)	4641 (92.4)
Others	1036 (7.3)	653 (7.2)	383 (7.6)
Sex			
Male	7100 (50.2)	5060 (55.5)	2040 (40.6)
Female	7048 (49.8)	4064 (44.5)	2984 (59.4)
Residence			
Urban	3669 (25.9)	2714 (29.7)	955 (19.0)
Rural	10,239 (72.4)	6231 (68.3)	4008 (79.8)
Missing	53 (0.4)	40 (0.4)	13 (0.3)
Educational level			
Illiterate	2414 (17.1)	1290 (14.1)	1124 (22.4)
Primary	6206 (43.9)	3753 (41.1)	2453 (48.8)
Junior/senior school	5203 (36.8)	3810 (41.8)	1393 (27.7)
College or above	325 (2.3)	271 (3.0)	54 (1.1)
Marital relationship	3.43 ± 0.82	3.56 ± 0.73	3.19 ± 0.90
Relationship with their children	3.64 ± 0.72	3.72 ± 0.67	3.50 ± 0.77
Air quality satisfaction	3.16 ± 0.82	3.21 ± 0.80	3.07 ± 0.85
Perceived health status	3.11 ± 1.02	3.35 ± 0.97	2.67 ± 0.95
Depression symptoms	8.21 ± 6.38	4.28 ± 2.83	15.35 ± 4.60
0–9	9124 (64.5)	-	-
10–19	4091 (28.9)	-	-
20–30	933 (6.6)	-	-

Spearman correlation analysis (Table 2) revealed that our key variables (marital relationships, relationships with their children, air quality satisfaction, perceived health status, and depression symptoms) was associated with each other significantly (*P* < 0.001). Depression symptoms correlated negatively with the other key variables, and the other variables correlated with each other positively.

**Table 2:** Correlation coefficients of key variables using Spearman correlation

Variables	1	2	3	4	5
1. Marital relationship	1				
2. Relationship with their children	0.463^***^	1			
3. Air quality satisfaction	0.250^***^	0.251^***^	1		
4. Perceived health status	0.165^***^	0.150^***^	0.096^***^	1	
5. Depression symptoms	−0.251^***^	−0.173^***^	−0.097^***^	−0.394^***^	1

Note: ^***^ *P* < 0.001

**Fig. 2:**
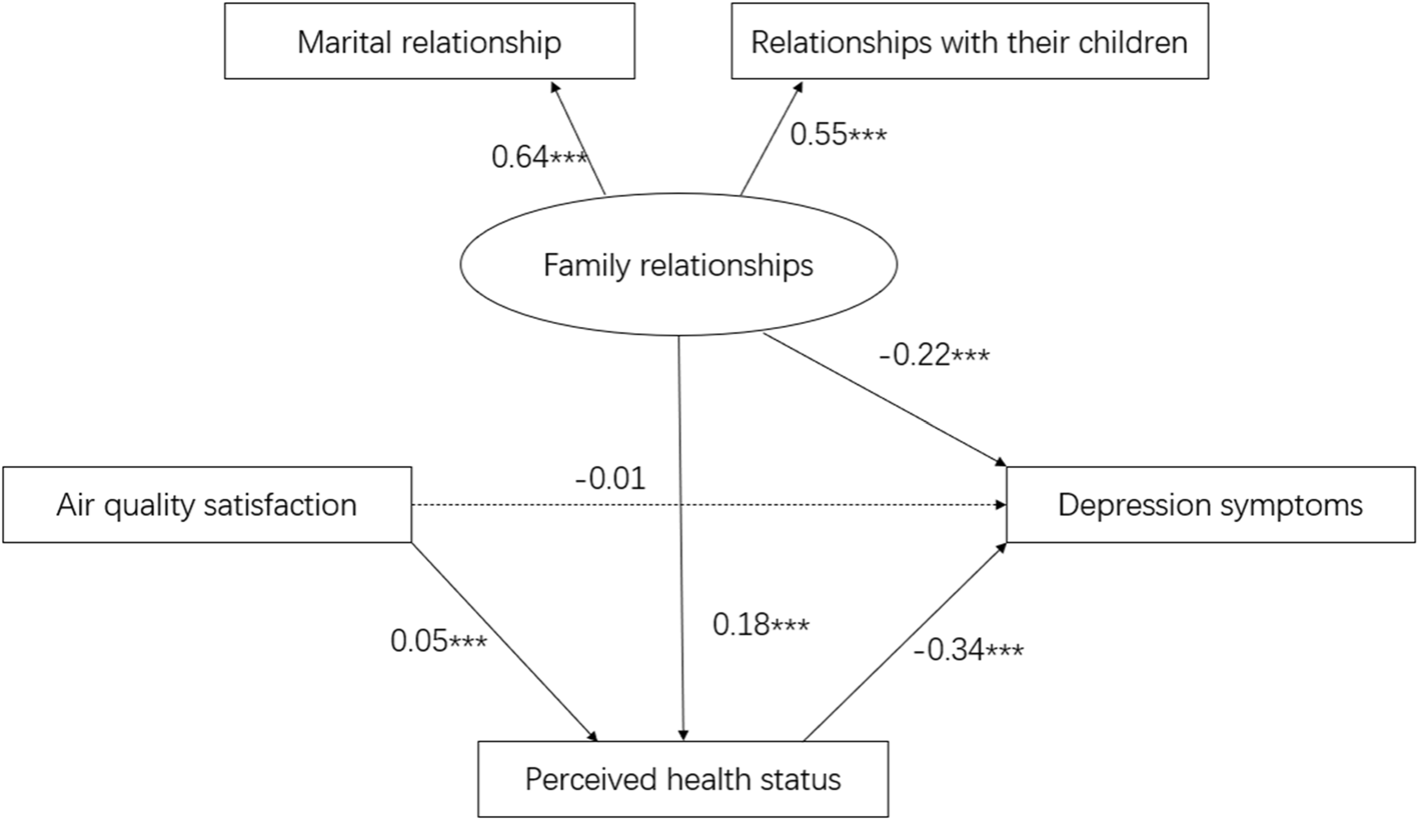
Association between air quality satisfaction, family relationships, and depression symptoms and the mediation role of perceived health status. (CMIN/DF = 4.87; NFI = 0.997; CFI = 0.998; TLI = 0.984; RF I = 0.980; RMSEA = 0.017). Note: The results are shown as the standardized β value, and the models were adjusted for sex and age; ^***^ *P* < 0.001

The mediation analysis is presented in Fig. 2. The final model showed a good model fit: χ^2^/df = 4.87, NFI = 0.997, CFI = 0.998, TLI = 0.984, RFI = 0.980, RMSEA = 0.017. The specific pathway effect of the key variables were as follows: air quality satisfaction to perceived health status (standardized beta = 0.05, *P* < 0.001), air quality satisfaction to depression symptoms (standardized beta =  −0.01, *p* = 0.23), perceived health status to depression symptoms (standardized beta = −0.34, *p* < 0.001), family relationship to perceived health status (standardized beta = 0.18, *p* < 0.001), family relationship to depression symptoms (standardized beta = −0.22, *p* < 0.001). In addition, the family relationship variables comprised marital relationships (standardized beta = 0.64, *p* < 0.001) and relationships with their children (standardized beta = 0.55, *p* < 0.001). Moreover, sensitivity tests using structural equation modeling-path analysis confirmed that the final model was stable and proved the mediation effect of perceived health status, with the coefficient size and *P*-value of each path being unchanged (See supplementary materials).

Total effects and indirect effects were confirmed using bootstrap methods, as shown in Table 3. In detail, the total standardized effect of family relationships on depression symptoms was −0.28 (95% CI: −0.31, −0.26), and air quality satisfaction on depression symptoms was −0.03 (95% CI: −0.05, −0.01). The total indirect standardized effect of family relationships on depression symptoms was −0.06 (95% CI: −0.07, −0.05), and the effect of air quality satisfaction on depression symptoms was −0.016 (95% CI: −0.02, −0.01). In conclusion, perceived health status full mediated the effect of air quality satisfaction on depression symptoms, and partly mediated the effect of family relationships on depression symptoms.

**Table 3:** Standardized total effect and indirect effect of the study variables on depression symptoms

Variables	Indirect effect		Total effect		
	β	95% CI		β	95% CI
Air quality satisfaction	−0.016	−0.02 ~ −0.01		−0.03	−0.05 ~ −0.01
Family relationship	0.06	−0.07 ~ −0.05		−0.28	−0.31 ~ −0.26

## Discussion

In this study, we revealed that marital relationships, relationships with their children, air quality satisfaction, perceived health status, and depression symptoms were associated with each other. Our study further confirmed that perceived health status mediated the association between air quality satisfaction and depression symptoms, and partly mediated the association between family relationships and depression symptoms. In addition, 35.5% (*n* = 5024) of the participants had depression symptoms. This ratio of depression was lower than the same survey conducted in 2013, suggesting a marked improvement in the healthcare of middle-aged and elderly adults in China [46]. Despite this success, the proportion of depressed middle-aged and elderly adults remains high, requiring targeted psychological intervention.

We also revealed that marital relationships, relationships with their children, air quality satisfaction, perceived health status, and depression symptoms were associated with each other. Although numerous studies have present positive or negative associations between them, no previous study reported the association between all variables [17, 33, 47]. This might be explained by the different design and main aims. For instance, a research group revealed the negative or positive association between depressive symptoms, well-being, and subjective pollution, but neglecting marital relationships and relationships with their children [33]. Besides, the correlation coefficients between our key variables are different and the size of the coefficients vary considerably, especially the association between air quality satisfaction and perceived health status, and depressive symptoms. The smaller coefficients of association between them revealed in this study can be attributed to the large sample size in this study. Similarly, previous large sample size international studies focusing on air pollution also almost reported little effect of air pollution [26, 48–50]. Thus, the associations between perceived health status, depressive symptoms, and air quality satisfaction existed, but were weak; therefore, these relationships require further research.

There was no direct impact of air quality satisfaction on depression symptoms, while air quality satisfaction correlated significantly and negatively with depression symptoms. Perceived health status played a full mediating role in the association between air quality satisfaction and depression symptoms. Previous studies theorized that air quality satisfaction was associated with perceived health status and in turn affected emotions, and hence depression [51, 52]. This study further confirmed this theory and provided quantitative evidence. In Li’s study, this mediation could be explained by the greater knowledge of air quality, leading to increased awareness of environmental pollution and the harmful consequence to the ecosystem, resulting in depression symptoms [37].

In this study, perceived health status was one part of psychological well-being, and family relationship satisfaction could affect depression symptoms under indirect mediation by perceived health status. Family relationships were an important factor of depression, and perceived health status partly mediated this effect. Family member relationships are associated with psychological well-being. A poor family member relationship could frustrate family well-being and the sense of belonging, perhaps impacting psychological well-being [31, 37], which could explain our results.

In this study, the standardized beta of the effect of perceived health status on depression symptoms was −0.34, which was the biggest coefficient of all pathways and was consistent with previous studies. Bea’s research showed that depression symptoms were the most powerful predictors of perceived health status, which was confirmed by a cohort study surveyed in Japan [35, 53]. Thus, perceived health status could be an essential index of depression symptoms among middle-aged and elderly adults. We believe that future studies should pay more attention to perceived health status.

There also were several limitations in this study. Firstly, this was a cross-sectional study, which might limit the causality of our key variables (family relationship satisfaction, air quality satisfaction, perceived health status, and depression symptoms). Secondly, key variables were measured by one sample item rather than using standardized scales (e.g., perceived health status), which limited the reliability and validity of the results. Thirdly, although the model P value was significant, the effect size was small, especially in the effect of air quality satisfaction on perceived health status (β = 0.05). Depression symptoms can be impacted by multiple societal, psychological, and biological factors. Consequently, we recommend that future studies should include other important factors (e.g., anxiety and suicidal ideation).

## Conclusion

In conclusion, this study explored the mechanism of the effects of family relationships, perceived health status, and air quality satisfaction on depression symptoms using a structural equation model. We found that marital relationships, relationships with their children, air quality satisfaction, perceived health status, and depression symptoms were associated with each other. Perceived health status fully mediated the association between air quality satisfaction and depression symptoms, and partly mediated the association between family relationships and depression symptoms. These findings highlighted the significance of the mediation effect, rather than the direct effect, of perceived health status on the association between air quality satisfaction or family relationships and depression symptoms. Our results have significance for the provision of psychological support services aiming to improve depression symptoms among the middle-aged and elderly population. In psychological support service programs, support service providers should pay more particular attention to improving the patient’s perceived health status.

## Supplementary Information

**Additional file 1: Figure S1. **Association between air quality satisfaction,family relationship and depression symptom and its mediation role of perceivedhealth status.

## Data Availability

The datasets generated and analysed during the current study are available in the CHARLS repository [http://charls.pku.edu.cn/].

## Abbreviations

CHARLS: The China Health and Retirement Longitudinal Study
CFI: Comparative fit index
TLI: Tucker-Lewis fit index
NFI: Normed fit index
RFI: Relative non-centrality index
RMSEA: the root mean-square error of approximation
CESD-10: the 10-item Center for Epidemiological Studies Depression Scale
