# Supplementary material for: Association between air quality satisfaction, family relationships, and depression symptoms among middle-aged and elderly chinese people: the mediation role of perceived health status
Source: BMC Public Health. 2022 Dec 27;22:2439. doi: 10.1186/s12889-022-14711-7 (PMC9795640; doi:10.1186/s12889-022-14711-7)
Supplement: Supplementary file 1 — Additional file 1: Figure S1. Association between air quality satisfaction,family relationship and depression symptom and its mediation role of perceivedhealth status. [file 12889_2022_14711_MOESM1_ESM.docx]

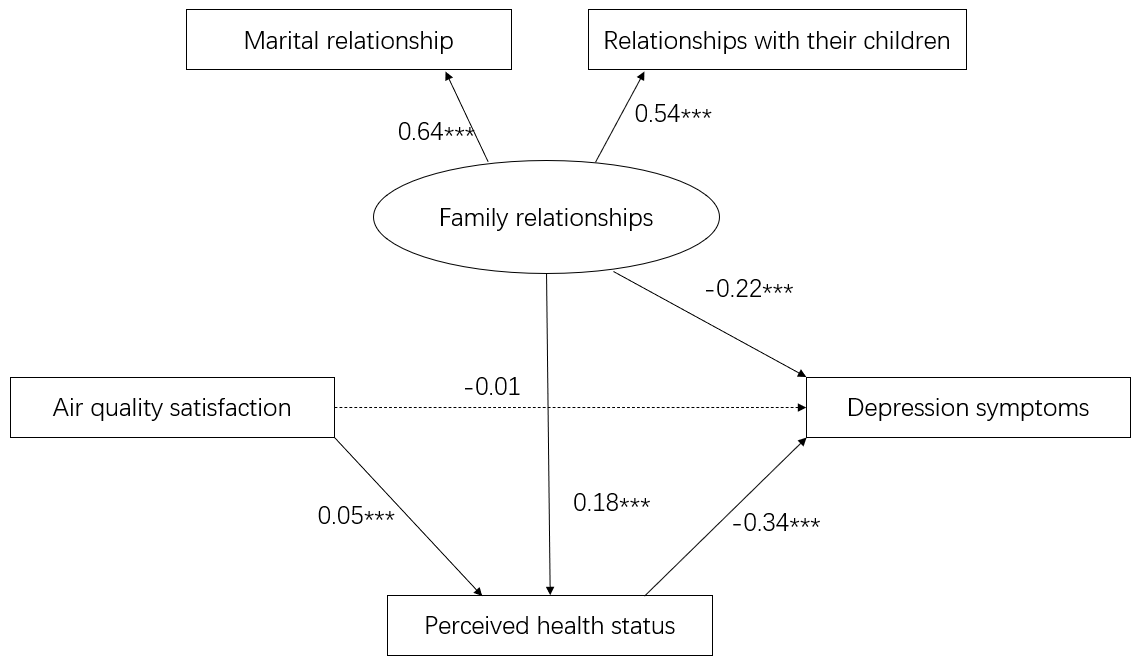


**Figure S1. Association between air quality satisfaction, family relationship and depression symptom and its mediation role of perceived health status.** (CMIN/DF=4.93 NFI=0.975 CFI=0.976 TLI=0.926 RFI=0.921, RMSEA=0.031).

Results were shown as the standardized β value of structural equation modeling-path models (SEM). Sex, age added educational level, ethnic, and residence were adjusted in the models; ^***^ was represented for P<0.001

**Sensitivity test:**

It is the results of sensitivity test of structural equation modeling-path analysis. In this structural equation modeling-path analysis, we controlled sex, age added educational level, ethnic, and residence characteristics. And the coefficient size and p value of each path remains unchanged, compared with the final model (See Figure 2). This result of sensitivity test confirmed that the final model was stability and proved the mediation effect of perceived health status.
